# Automatization of CT Annotation: Combining AI Efficiency with Expert Precision

**DOI:** 10.3390/diagnostics14020185

**Published:** 2024-01-15

**Authors:** Edgars Edelmers, Dzintra Kazoka, Katrina Bolocko, Kaspars Sudars, Mara Pilmane

**Affiliations:** 1 Institute of Anatomy and Anthropology, Rīga Stradiņš University, LV-1010 Riga, Latvia; dzintra.kazoka@rsu.lv (D.K.); mara.pilmane@rsu.lv (M.P.); 2 Department of Computer Graphics and Computer Vision, Riga Technical University, LV-1048 Riga, Latvia; katrina.bolocko@rtu.lv; 3 Institute of Electronics and Computer Science, LV-1006 Riga, Latvia; sudars@edi.lv

**Keywords:** radiology, artificial intelligence, computer vision, semantic segmentation, annotation

## Abstract

The integration of artificial intelligence (AI), particularly through machine learning (ML) and deep learning (DL) algorithms, marks a transformative progression in medical imaging diagnostics. This technical note elucidates a novel methodology for semantic segmentation of the vertebral column in CT scans, exemplified by a dataset of 250 patients from Riga East Clinical University Hospital. Our approach centers on the accurate identification and labeling of individual vertebrae, ranging from C1 to the sacrum–coccyx complex. Patient selection was meticulously conducted, ensuring demographic balance in age and sex, and excluding scans with significant vertebral abnormalities to reduce confounding variables. This strategic selection bolstered the representativeness of our sample, thereby enhancing the external validity of our findings. Our workflow streamlined the segmentation process by eliminating the need for volume stitching, aligning seamlessly with the methodology we present. By leveraging AI, we have introduced a semi-automated annotation system that enables initial data labeling even by individuals without medical expertise. This phase is complemented by thorough manual validation against established anatomical standards, significantly reducing the time traditionally required for segmentation. This dual approach not only conserves resources but also expedites project timelines. While this method significantly advances radiological data annotation, it is not devoid of challenges, such as the necessity for manual validation by anatomically skilled personnel and reliance on specialized GPU hardware. Nonetheless, our methodology represents a substantial leap forward in medical data semantic segmentation, highlighting the potential of AI-driven approaches to revolutionize clinical and research practices in radiology.

## 1. Introduction

Recent advancements in artificial intelligence (AI) have revolutionized numerous sectors, including automotive engineering, economics, finance, and particularly medical diagnostics and treatment planning. AI, a pivotal branch of computer science, involves designing algorithms capable of mimicking human tasks, thereby facilitating in-depth learning, remote teaching, prompt feedback, innovative assessment, and efficient data storage in education [1]. According to Saga et al. [2], AI’s precision and wide-ranging benefits have attracted extensive research interest. In medical contexts, AI has proven invaluable in managing large datasets, aiding in clinical decision-making, and enhancing the accuracy of medical practice [3,4,5,6,7,8,9]. Specifically, AI-powered tools have become integral in image-guided systems for surgical planning and simulation, leveraging advanced visualization technologies [10].

Moreover, the evolution of imaging techniques has markedly improved the visualization and identification of anatomical structures. AI’s application in anatomy education promises significant advancements, potentially enhancing both student learning and instructor teaching methodologies [11,12]. The scalability and speed of AI technologies offer unprecedented opportunities in educational settings [13]. In medical data analysis, AI’s precision is instrumental in CT and MRI scans, enabling the detailed detection of complex anatomical structures. This precision aids in various medical applications, from surgery planning to patient care. AI also plays a crucial role in developing and utilizing 3D anatomical models for educational purposes, enhancing students’ comprehension of human anatomy [14,15]. Visual representations in medical imaging are vital for professionals, researchers, educators, students, and patients. AI has significantly advanced medical data annotation methods, including in the study of human bone structures. AI algorithms efficiently process and update large datasets, creating detailed and accurate images for medical analysis [14,15].

This paper presents a semi-automatic workflow for the semantic segmentation of human spine CT data using AI tools. This method greatly reduces the time and resources required for manual segmentation, thereby offering significant benefits in AI and healthcare applications and proving effective upon testing. The workflow includes volume stitching for multiple series, AI-driven semantic segmentation, validation of anatomical correctness, and practical applications of segmented radiological data. Section 2 details the materials and methods, Section 3 presents the results, Section 4 analyzes the methodology and concludes the paper, discussing potential future research directions.

### 1.1. Performance Semantic Segmentation

This section outlines a method for performing semi-automatic semantic segmentation of computed tomography (CT) data, utilizing AI-based tools. The workflow is methodically structured into four distinct stages, two of which are mandatory and two optional, depending on the specific requirements of the dataset.

**Volume Stitching (Mandatory in Certain Cases)**: This step is essential in instances where multiple series are involved, such as when the CT data encompasses separate scans of the cervical region and thoraco-abdominal areas. The stitching process integrates these series into a cohesive volume, facilitating a more comprehensive analysis.

**Semantic Segmentation with AI (Mandatory):** At this core stage, AI-driven instruments are employed to segment the CT data semantically. The AI tools identify and delineate various anatomical structures within the scans, streamlining the segmentation process.

**Validation of Segmentation Anatomical Correctness (Mandatory):** This critical step involves the verification of the AI-segmented data for anatomical accuracy. It ensures that the segmentation aligns correctly with established anatomical standards, thereby maintaining the precision and reliability of the results.

**Post-Processing (Optional):** In cases where further refinement or adjustments are necessary, post-processing can be applied. This step allows for the fine-tuning of the segmented data, addressing any specific needs or discrepancies that may arise from the initial AI segmentation.

### 1.2. Volume Stitching

In radiological imaging, particularly for semantic segmentation, the integration of individual volumes is paramount. This is especially true for complex anatomical structures like the spine, where a comprehensive and uninterrupted view is essential. The vertebral column extends from the cervical region at the top to the thoraco-abdominal region at the bottom. If these areas are scanned separately, the resulting images may be fragmented, failing to provide a holistic view of the spine’s anatomy.

Semantic segmentation, a process where each pixel in an image is classified into specific categories such as different tissues or structures [16], relies heavily on the completeness of the data. By stitching together separate volumes from cervical and thoraco-abdominal scans, we ensure that the segmentation algorithms have a full representation of the spine. This holistic view is crucial for achieving accurate and reliable segmentation outcomes.

Furthermore, different scanning protocols for cervical and thoraco-abdominal regions often lead to variations in image quality, resolution, or contrast [17]. By merging these distinct volumes, we create a uniform dataset, which is critical for consistent analysis. This uniformity significantly reduces the likelihood of errors or inconsistencies that can arise during the segmentation process. Moreover, working with a single, unified dataset, as opposed to multiple disjointed ones, greatly streamlines the analysis process, enhancing efficiency and accuracy.

### 1.3. Semantic Segmentation with AI

Semantic segmentation, a critical process in medical radiology, involves classifying each pixel in an image into specific classes, thereby extracting detailed anatomical and pathological information. This technique is instrumental in diagnosing diseases, planning treatments, and monitoring disease progression. Despite its importance, the implementation of semantic segmentation in medical radiology encounters several significant challenges.

One primary challenge is the inherent complexity of medical images. Modalities like MRIs, CT scans, and X-rays reveal detailed internal structures, where the diverse appearances of tissues and overlapping structures can complicate the segmentation process. Additionally, images are often subject to noise, artifacts, and distortions due to factors like patient movement, limitations of imaging devices, or specific scanning parameters [18,19]. These imperfections can lead to inaccurate segmentations. Another obstacle is the variability among patients. Factors such as age, genetics, and health conditions result in wide anatomical differences, often necessitating extensive customization or manual adjustments in standardized segmentation models. Further complexity arises from the high dimensionality of medical imaging; CTs and MRIs produce volumetric, 3D data, adding to the segmentation challenges.

A significant hurdle in semantic segmentation is the scarcity of accurately annotated data. Supervised learning models require extensive labeled datasets for training [20]. However, obtaining precise annotations from experienced radiologists is a resource-intensive and time-consuming task. Class imbalance in medical images, where crucial features like tumors may occupy a small portion of the image, can skew algorithms towards more dominant classes, leading to segmentation inaccuracies [21].

Inter-rater variability in medical image annotations poses another challenge. Even among expert radiologists, discrepancies in image interpretation and annotation are common [22]. This variability complicates the establishment of a consistent ground truth for training models. In certain applications, such as image-guided surgeries, real-time semantic segmentation is required, adding to the complexity.

Lastly, the issue of model generalization is a significant concern. Models trained with data from one medical institution or a specific imaging device may not perform well with data from different sources, due to variations in imaging protocols, patient demographics, or device characteristics [23]. This highlights the need for adaptable and robust models capable of handling diverse datasets.

The recent advancements in machine learning (ML) and deep learning (DL) algorithms have markedly increased the adoption of artificial intelligence (AI) in the medical field [24]. These developments, coupled with powerful new imaging modalities provided by advanced scanners, present unique challenges in visualization. Future healthcare technologies, driven by these advancements, are expected to enhance the quality of medical imaging while reducing associated costs, thereby simplifying the extraction and optimal combination of individual data [25].

Computer-aided platforms, leveraging these technological advancements, assist medical professionals in making critical clinical decisions related to diagnosis, disease tracking, and prognosis [26,27,28]. Medical image creation, tailored to individual patients’ medical data, enables professionals to visualize and detect specific manifestations of diseases, enhancing personalized medical care. AI algorithms play a crucial role in diagnosing diseases, planning effective treatment strategies, and monitoring treatment outcomes. These algorithms can analyze medical imaging data to create detailed illustrations of bone dislocations, fractures, or tumors, specifying their location, size, shape, and characteristics. Moreover, AI has demonstrated its capability to surpass human performance in certain tasks, such as image segmentation [29]. It can process a wide array of data types in the medical context, including outputs from imaging scanners, sensor data, or patient metadata. Modern computers, equipped with DL algorithms, are adept at semantic labeling and image classification. In medical data analysis, these algorithms focus on identifying anatomical landmarks, geometric descriptors, centerlines of structures, shape, deformation, and fiber orientation, thereby providing remarkable diagnostic accuracy for various diseases [30].

New methodologies in AI have been developed for the identification and classification of objects in medical images [31,32]. The creation of better methods and standards for designing, implementing, and evaluating clinical AI-enabled instruments is crucial to ensure their effectiveness and value to end users [33]. These algorithms, trained on large datasets, can predict outcomes on new data without explicit programming [34]. Current computer vision algorithms excel at identifying patterns in digital data, achieving human-level accuracy in object detection.

Image segmentation, a fundamental procedure in many medical imaging applications, involves delineating regions within images that are occupied by objects of interest [35]. Traditional segmentation methods, which often rely on areas and edges, face limitations due to factors such as non-uniform grayscale, individual differences, and the presence of artifacts and noise in images [36]. The advent of deep learning (DL) has brought about advanced architectures and feature extraction mechanisms that significantly enhance the segmentation of deformed anatomy [37], thereby improving the accuracy of disease diagnosis and minimizing redundant computations. Automatic segmentation of bones is a crucial step in deriving quantitative markers for accurate clinical diagnosis and in developing computer-aided decision support systems. In modalities like computed tomography (CT) and magnetic resonance imaging (MRI), understanding the pathology and observing changes in the anatomical structures, shape, size, and texture of bones are vital for initial disease diagnosis and monitoring its progression. For instance, in spine image analysis and 3D spine reconstruction applications, accurately locating and segmenting vertebrae from CT spinal images is fundamental [38]. Accurate bone segmentation provides a stable structural reference for both inter and intra-patient registration and internal organ localization, facilitating the automatic segmentation of internal organs [39]. However, despite the relative ease of visual observation of bones in CT images, challenges such as low signal-to-noise ratio, insufficient spatial resolution, and indistinct image intensity between spongy bones and soft tissues make the precise segmentation of individual bones a complex task [40]. Accurately segmenting the spine into individual vertebrae is crucial for diagnosing spine-related illnesses, especially for detecting and classifying bone damage, fractures, lesions, and tumors [41,42,43].

### 1.4. Validation of Segmentation Anatomical Correctness

To maintain the anatomical accuracy of the model, it is cross-referenced with credible academic references. Furthermore, the use of expert and accredited medical virtual applications is recommended. Complete Anatomy serves as a prime example of such tools, providing an accurate and intuitive interface for manipulating anatomical models. Developed by 3D4Medical under the aegis of Elsevier, Complete Anatomy stands as a sophisticated educational 3D anatomy platform, with the company’s expertise in the development of medical products dating back to 2009 [44,45].

### 1.5. Practical Use of Segmented Radiological Data

Semantically segmented medical datasets have become fundamental in advancing medical research and technology. Their integration into artificial intelligence (AI) and 3D technologies heralds a new era of opportunities and breakthroughs.

In medical AI, these datasets are indispensable. AI algorithms, trained on semantically segmented data, have transformed diagnostic processes by precisely identifying and classifying abnormalities [46]. This advancement aids radiologists and clinicians in early and accurate diagnosis, paving the way for personalized medicine. Furthermore, AI models utilizing these datasets can perform predictive analyses, anticipating disease progression, patient outcomes, and treatment efficacy [47]. Automated segmentation, another critical application, allows AI to process intricate medical images efficiently, reducing manual labor and ensuring uniformity in results [48,49,50].

Three-dimensional technologies have significantly benefited from these datasets, enabling the creation of detailed anatomical reconstructions [51]. Surgeons utilize these 3D models for meticulous procedural planning, enhancing patient outcomes and optimizing surgical times. Additionally, the integration of these datasets into virtual and augmented reality platforms enhances medical training and patient education, providing immersive experiences and real-time surgical guidance [52].

Beyond AI and 3D applications, semantically segmented datasets are pivotal in telemedicine [53]. They facilitate real-time sharing and analysis, ensuring access to expert care regardless of location. These datasets are at the forefront of contemporary medical innovation, enhancing current practices and setting the stage for future advancements.

In clinical contexts, computer-aided and semi-automated segmentation techniques are crucial for interpreting CT and MRI images for bone pathologies. Correct vertebra segmentation allows for monitoring the progression of diseases under treatment. Traditional manual segmentation is time-consuming, requiring an in-depth understanding of anatomy and extensive effort; for instance, segmenting a vertebral column can take up to 24 h, depending on data quality and scanning protocols. This duration may double for novices. Our proposed methodology enables non-specialists to perform initial segmentation of general, non-pathological structures semi-automatically. This process provides a basis for adjustments, allowing initial segmentation by AI, followed by manual validation and refinement against literature sources and anatomical standards.

## 2. Materials and Methods

The 3D Slicer software (version: 5.4; slicer.org (accessed on 30 August 2023) [54] was used in this work. It is important to highlight that the program can be easily downloaded and used at no cost, being an open-source software.

The dataset used in this research comprised 250 patient CT scans sourced from Riga East Clinical University Hospital.

In cases where it is necessary to merge multiple series in order to segment the whole anatomical structure, the steps below should be followed.

### 2.1. Methodology for Series Merging (Optional)

Data import

1. Start the 3D Slicer software.;
2. Access the DICOM database;
3. Choose the appropriate patient;
4. Identify two series intended for later merging;
5. Import the identified series.

Creation of region of interest (ROI)

1. Activate the Crop Volume module;
2. Under Input Volume, choose a series;
3. Under Input ROI, opt for Create ROI. The resulting volume will be named Crop Volume ROI;
4. Click the Center View button in the 3D visualization window;
5. Select the Fit to Volume option;
6. Resize the ROI to ensure both loaded volumes, presented as two individual entities, fit within this new ROI.

The process of stitching two volumes

1. Access the Stitch Volume module;
2. Under Stitched Volume ROI, select the Crop Volume ROI you previously created;
3. For Original Volume 1, choose the primary volume to which the secondary volume will be appended;
4. For Original Volume 2, select the other loaded volume;
5. Click the Create Stitched Volume button.

The Stitch Volume function is designed to create a unified composite image from multiple image volumes. It operates by selecting specific images and merging them within a user-defined rectangular region of interest (ROI). This function meticulously handles the trimming and fusion of overlapping areas in the volumes, dividing each area based on its proximity to the midpoint of the overlap.

Key to the process is the preservation of image resolution. The function precisely adopts the resolution of the initial input volume. To maintain image fidelity, nearest-neighbor interpolation is applied, which accounts for minor shifts in voxel placement. In situations where the image volumes have varying resolutions, windowed sync interpolation is utilized to achieve a uniform resolution across the merged image.

For areas outside the scope of the original volumes, the function assigns a default voxel value of zero. This assignment ensures clear demarcation of the merged areas from the non-covered regions. The process concludes with the generation of a consolidated image, confined within the defined ROI and matching the resolution of the first input volume.

### 2.2. Methodology for Segmentation

The creation of semantic mask|TotalSegmentator module

1. For Input Volume, choose the volume you have loaded;
2. In Segmentation Tasks, opt for Total;
3. Click the Apply’ button.

Deletion of unnecessary data

4. Remove all items, retaining only the volume used in the prior step and the desired semantic segmentations of anatomical structures.

Masks validation and correction

5. When smoothing is necessary:
  1. For the Smoothing Method, select Median;
  2. Set the Kernel Size to 3.00 mm;
  3. Toggle the Apply to Visible Segments option to Enable.
6. Verify the morphological accuracy of segmented structures using references from literature or digital platforms. If required, make adjustments utilizing the Draw, Paint (with the Sphere Brush feature activated), Erase, and Scissors tools.
7. If there is a segmentation error where a part of one structure is identified as a segment of another bone, follow these steps to merge and rectify:
  1. In the Data module, duplicate the segment that contains a section of the incorrect bone;
  2. Utilize the Scissors tool from the Segment Editor module to remove everything except the mislabeled structure;
  3. Using the Logical Operators tool, integrate the two segments of a single structure using the Add operation.

Data export

8. Save the volume in *.nrrd* file format;
9. Save segmentation for the volume in *seg.nrrd* file format.

## 3. Results

The methodology we discuss has been rigorously validated by applying it to a dataset of 250 patient CT scans from Riga East Clinical University Hospital. This validation specifically involved the semantic segmentation of the vertebral column, with a keen focus on accurately registering and naming each individual vertebra.

In selecting our patient cohort, we implemented a meticulously designed criterion to ensure a demographically balanced sample in terms of age and sex, thus enhancing the generalizability of our study’s findings. A pivotal inclusion criterion was the structural integrity of the vertebral column. We exclusively analyzed scans where the vertebrae were free from severe dispositions or deformities to minimize potential confounding factors that could bias our results.

**Figure 1:**
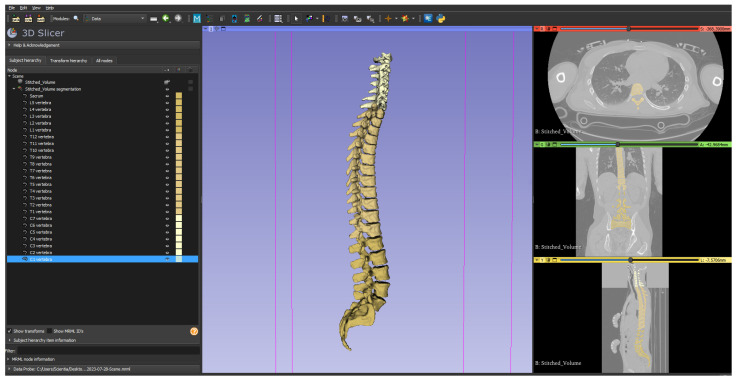
Semantically segmented vertebral column serving as proof of concept of the presented methodology (in total, 25 bones from C1 till sacrum combined with coccyx).

The core of our workflow, aside from the optional step of volume stitching to create a single comprehensive volume, was the segmentation of the vertebral column. This involved the detailed semantic segmentation of all 25 bones in the vertebral column, including C1 to C7 (cervical), T1 to T12 (thoracic), L1 to L5 (lumbar), and the sacrum combined with the coccyx. An illustrative example of a semantically segmented vertebral column, as executed following our presented methodology, is depicted in Figure 1.

**Figure 2:**
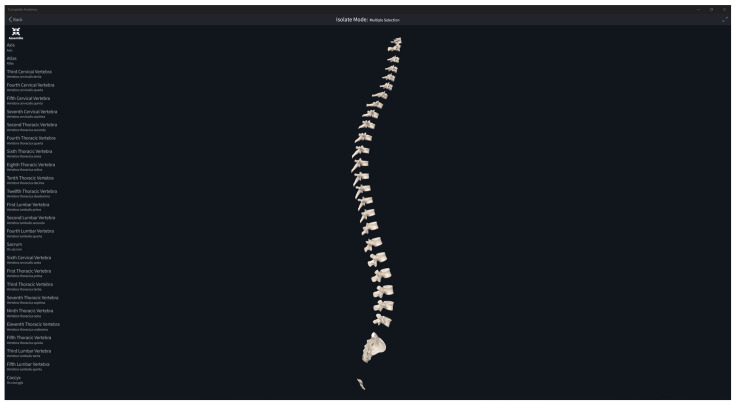
Reference digital models of vertebras used during the validation methodology from Complete Anatomy software (ver. 10.0.1) [44].

The segmentation was conducted under the supervision of an experienced radiologist and an anatomy professor, ensuring accuracy and adherence to established medical standards. All the created masks have been validated and corrected according to the methodology by comparing all the semantically segmented bones with the reference anatomy from the literature (Figure 2).

In our analysis, we identified instances of false registration anomalies (in 62 cases), a significant error in which two separate vertebrae were mistakenly identified and registered as a single anatomical entity. This misregistration presents a considerable source of error, adversely affecting both the qualitative and quantitative assessments of spinal structure.

**Figure 3:**
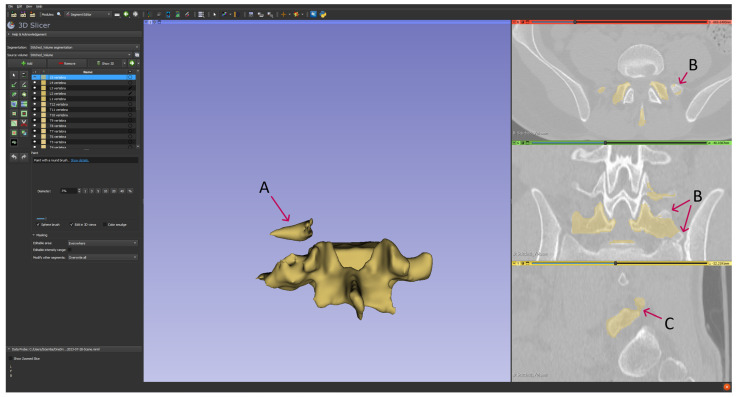
Illustrative examples of artifacts in anatomical segmentation. (A) Improper registration: A segment of the L4 vertebra is inaccurately identified as part of the L5 vertebra. (B) Omission of critical regions: This artifact represents the absence of specific anatomical regions that should have been included in the segmentation. (C) False registration: Soft tissues are incorrectly registered as constitutive elements of the L5 vertebra, compromising the integrity of the segmented structure.

Moreover, we observed segmentation inaccuracies where the delineated region either exceeded or fell short of the intended anatomical limits (in all cases). In some cases, the segmentation extended into adjacent tissues, while in others, it failed to include parts of the target structure. Such deviations result in a misrepresented portrayal of the spine’s true morphology, as exemplified in Figure 3.

These artifacts not only undermine the data’s accuracy but also present substantial challenges in clinical or research contexts that depend on precise anatomical demarcation. Ensuring the correctness of vertebral identification and maintaining the integrity of anatomical boundaries are crucial for the reliability and applicability of our findings in subsequent medical analyses and applications.

The successful conclusion of our study resulted in the creation of a composite volume, which effectively integrated two critical anatomical regions: the cervical and the thoraco-abdominal areas. This integration was complemented by their corresponding segmentation masks. A notable aspect of our data storage approach was the adoption of the Nearly Raw Raster Data (.NRRD) file format, diverging from the traditional Digital Imaging and Communications in Medicine (DICOM) format.

This deliberate choice to utilize the .NRRD format was driven by its several advantages, particularly in the context of machine learning applications. The .NRRD format facilitates faster data loading speeds, a crucial factor in efficiently processing large datasets in AI-driven research. This efficiency is especially beneficial when dealing with the complex, multi-region datasets like ours, where streamlined data handling can significantly enhance computational performance and analysis throughput.

## 4. Discussion

Our methodology employs AI in an easy-to-use format, providing operators with a graphical user interface (GUI). All necessary components related to different Slicer modules are pre-installed within the program, eliminating the need for external downloads.

However, a significant limitation of this methodology is the need for manual validation of all segmented anatomical structures. This process requires a high level of anatomical expertise and introduces potential sources of error and subjectivity. The segmentation process is prone to inaccuracies such as the omission of critical regions, incorrect merging of different anatomical regions, and imprecise registration of structures. For example, two adjacent vertebrae might be inaccurately registered as a single entity, affecting data integrity. Pathological anomalies and anatomical variations add complexity to the segmentation task, requiring both AI and human validators to uphold morphological precision and methodological robustness. The lack of quantitative benchmarks and metric evaluation for AI-segmented structures further complicates the validation process.

Additionally, the segmentation capabilities are limited by the TotalSegmentator software (ver. 2.0.4), which supports only a specific range of anatomical structures, thereby restricting the method’s applicability in broader anatomical studies [55,56].

This methodology introduces an enhanced approach to the annotation of radiological data. Its distinct advantage lies in its broad applicability to a variety of anatomical structures, making it a valuable tool in AI-driven projects, especially in the area of medical data segmentation. A notable feature of this methodology is its inclusive design, which expands the range of annotators beyond traditional radiological professionals. This inclusivity accelerates the data annotation process, enabling quicker completion of projects and resulting in efficient use of resources. Furthermore, for specialists in segmentation tasks, this approach offers a significant reduction in the time required, enhancing overall productivity.
